# Identifying causal rare variants of disease through family-based analysis of Genetics Analysis Workshop 17 data set

**DOI:** 10.1186/1753-6561-5-S9-S21

**Published:** 2011-11-29

**Authors:** Wai-Ki Yip, Gourab De, Benjamin A Raby, Nan Laird

**Affiliations:** 1 Department of Biostatistics, Harvard School of Public Health, 655 Huntington Avenue, Building II, Boston, MA 02115, USA; 2 Brigham and Women Hospital, 75 Francis Street, Boston, MA 02115, USA

## Abstract

Linkage- and association-based methods have been proposed for mapping disease-causing rare variants. Based on the family information provided in the Genetic Analysis Workshop 17 data set, we formulate a two-pronged approach that combines both methods. Using the identity-by-descent information provided for eight extended pedigrees (*n* = 697) and the simulated quantitative trait Q1, we explore various traditional nonparametric linkage analysis methods; the best result is obtained by assuming between-family heterogeneity and applying the Haseman-Elston regression to each pedigree separately. We discover strong signals from two genes in two different families and weaker signals for a third gene from two other families. As an exploratory approach, we apply an association test based on a modified family-based association test statistic to all rare variants (frequency < 1% or < 3%) designated as causal for Q1. Family-based association tests correctly identified causal single-nucleotide polymorphisms for four genes (*KDR*, *VEGFA*, *VEGFC*, and *FLT1*). Our results suggest that both linkage and association tests with families show promise for identifying rare variants.

## Background

In contrast to the common variant/common disease hypothesis that dominated the era of linkage-disequilibrium-based genome-wide association studies (GWAS), there is increasing awareness that rare variants of modest to large individual effect contribute to disease liability and may explain a substantial proportion of the so-called missing heritability of common traits. There is therefore great interest in developing statistical methods to detect rare causal variants. Rare variant analysis is complicated by several unique challenges related to sequencing-based uncertainties in variant calling, the large search space of rare variants, and the inherently low carrier rate frequencies of these variants. It has been theorized that both linkage and family-based analysis work well in analyzing rare variants [1,2]. Combining both approaches may provide a powerful strategy for identifying rare variants.

## Methods

The Genetic Analysis Workshop 17 (GAW17) data set was developed to model a real-world rare variant screen using data generated from a mini-exome scan [3]. The genotype data correspond to 24,487 variants (in 3,205 genes) derived from low-coverage sequence data provided from the 1000 Genomes Project. In our analysis, we use the simulated family-based sample of eight three-generation pedigrees (697 individuals). The founders of these pedigrees are a random sample of 202 individuals selected from the population-based sample. As a result, only four of the nine causal genes have low-frequency causal single-nucleotide polymorphisms (SNPs) in the family data. In our linkage analysis and initial family-based association test (FBAT) analysis, we average the 200 replications of the Q1 phenotype to maximize power. Detailed information about the pedigrees is shown in Table 1.

**Table 1:** Pedigree information based on the combined sample

Pedigree number	Number of nuclear families	Number of affected sibs	Total number of sib pairs	Number of affected sib pairs
0	23	22	86	5
1	29	26	100	8
2	26	29	90	4
3	20	19	74	1
4	20	18	73	2
5	20	20	73	7
6	36	48	128	34
7	20	18	73	1
Total	194	200	697	62

### Linkage analysis

Our initial goal is to evaluate a variety of linkage-based approaches using the within-family identity-by-descent (IBD) information provided. In the absence of knowledge regarding the disease model, we restricted our evaluation to nonparametric methods so as to maximize power [4]. We evaluated several approaches that consider either all sib pairs (SPs) or only affected sib pairs (ASPs), including goodness-of-fit, mean, and trend test using ASPs and the Haseman-Elston and modified Haseman-Elston regressions using all SPs [4-7]. We also used the Haseman-Elston regression with Q1. Because this proved to be the most powerful, we restrict our reporting to this approach.

### Family-based association test

The resolution of linkage analysis is limited by the number of informative meioses within each pedigree (a function of pedigree structure and randomness). We therefore consider family-based association methods to facilitate fine mapping of linked regions. The association test is based on a modified FBAT [8] statistic as follows: Suppose we have *i* = 1, …, *N* independent trios and *M* rare variants in a given gene. We apply the test to markers using a defined rare variant allele frequency threshold (<1% and 3% are illustrated). The cutoff is arbitrary and deserves further exploration. The test statistic has the following numerators:(1)

where *T_i_* is the trait, *X_i_* is the observed number of rare variant alleles among the offspring for the *i*th family, *μ* is the trait offset (typically the mean for measured traits), and *P_i_* is the parental genotype corresponding to the *i*th family. The numerator is the sum of individual numerators of each of the FBAT statistics for all *M* SNPs. It represents the contributions for all families over all variants in a given gene to the new FBAT statistics. The test statistic *W*/[Var(*W*)]^1/2^ is a *Z*-statistic that can be used to test against a one-sided or two-sided alternative.

The variance of *W* has a complicated expression. Even if we assume that the nuclear families within a pedigree are independent, estimating the covariance structure between the SNPs for each family is difficult because of the presence of linkage disequilibrium between variants. For the purpose of this project, we use the empirical variance as the denominator, which gives:(2)

Instead of trios, we can extend the numerator by summing contributions over all nuclear families in all pedigrees:(3)

where the summand corresponds to the *l*th offspring of the *i*th nuclear family in the *k*th pedigree. We can compute the empirical variance in two different ways, by treating either the pedigrees:(4)

or the nuclear families:(5)

as independent units, where the term in braces in expression (4) or (5) is the contribution of the pedigree or the nuclear family. The choice of assumption has important implications for test performance. Assuming that nuclear families are independent gives a biased estimate of the variance if indeed phenotypic correlation exists between nuclear families within a pedigree. Alternatively, assuming that pedigrees are independent gives a conservative estimate of the variance when only a small number of pedigrees are studied, as in the GAW17 family data. This test can also be extended to nuclear families with missing parents by conditioning on a sufficient statistic for transmission instead of parental genotype.

## Results

### Comparison of linkage-based approaches

We observe striking differences in the performance of the various linkage-based approaches evaluated. Any linkage method that aggregated results across pedigrees failed to identify any of the causal genes among the top candidates. In contrast, when genetic heterogeneity was considered by performing pedigree-stratified analysis, some of the causal genes were identified. The results are summarized in Table 2. *KDR* (*p* = 2.0 × 10^−8^) is the top gene and is most significant in one pedigree; *VEGFA* (*p* = 1.4 × 10^−5^) is among the top significant genes in another pedigree; and *FLT1* (*p* = 5.4 × 10^−3^ and 1.0 × 10^−3^) shows up as the top gene in two other pedigrees, but the signal seems to be significantly weaker.

**Table 2:** Top candidate genes from separate pedigrees

Pedigree 1	*p*-value	Pedigree 3	*p*-value	Pedigree 4	*p*-value	Pedigree 5	*p*-value
*GPR115*	0.000004	*KDR*	0.00000002	*EPHA6*	0.0052	*PIBF1*	0.0003
*C6orf130*	0.000013	*KIT*	0.00000002	*GPR128*	0.0052	*CCNA1*	0.0009
*GUCA1B*	0.000013	*LNX1*	0.00000002	*OR5K1*	0.0052	*CYSLTR2*	0.0009
*KIAA0240*	0.000013	*PDGFRA*	0.00000002	*OR5K2*	0.0052	*DGKH*	0.0009
*MEA1*	0.000013	*SGCB*	0.00000002	*OR5K3*	0.0052	*DNAJC15*	0.0009
*PPP2R5D*	0.000013	*SPATA18*	0.00000002	*OR5K4*	0.0052	*ELF1*	0.0009
*PRPH2*	0.000013	*PPAT*	0.00000045	*ST3GAL6*	0.0052	*FNDC3A*	0.0009
*PTK7*	0.000013	*SPINK2*	0.00000045	*B3GALTL*	0.0054	*FREM2*	0.0009
*RGL2*	0.000013	*GUF1*	0.00005598	*BRCA2*	0.0054	*HTR2A*	0.0009
*SLC26A8*	0.000013	*NFXL1*	0.00005598	*FLT1*	0.0054	*NUFIP1*	0.0009
*TAF11*	0.000013	*CHRNA9*	0.00047549	*LOC650794*	0.0054	*P2RY5*	0.0009
*TBCC*	0.000013	*NSUN7*	0.00047549	*SGCG*	0.0054	*RB1*	0.0009
*TFEB*	0.000013	*RHOH*	0.00047549	*TNFRSF19*	0.0054	*RCBTB2*	0.0009
*ZNF76*	0.000013	*LZTR1*	0.00167619	*ZMYM2*	0.0054	*TRPC4*	0.0009
*NFKBIE*	0.000014	*SCARF2*	0.00167619	*ZMYM5*	0.0054	*FLT1*	0.0010
*RUNX2*	0.000014	*SDF2L1*	0.00167619	*NFKBIZ*	0.0092	*STARD13*	0.0011
*SUPT3H*	0.000014	*TOP3B*	0.00167619	*STARD13*	0.0208	*B3GALTL*	0.0013
*VEGFA*	0.000014	*JMJD2C*	0.00242110	*ATP10A*	0.0213	*BRCA2*	0.0013
*HFE*	0.000019	*PTPRD*	0.00242110	*ADCY5*	0.0229	*LOC650794*	0.0015
*HIST1H2AA*	0.000019	*KIAA1432*	0.00303729	*ADPRH*	0.0229	*SGCG*	0.0015

Linkage analysis results of top candidate genes by regressing the square of the difference of Q1 against IBD for all sib pairs in a pedigree.

### Fine mapping result

To assess the performance of our modified FBAT statistic, we first screened all variants with a frequency less than 1% for association using a univariate application of the standard FBAT statistic (considering individual variants separately). We found that, with the exception of one disease-causing variant (C4S1884), all the variants demonstrated trends of association (at α = 0.05), although none reached significance after adjustment for multiple tests. We next applied our modified FBAT, performing gene-based tests of all rare variants with frequencies less than 1% or less than 3%. The *p*-values corresponding to the true causal genes are summarized in Table 3. Of the four genes with causal rare variants in the family data, we detected association (*p* < 0.01) for three genes (*VEGFA*, *VEGFC*, and *FLT1*), and for the fourth gene (*KDR*), significance was achieved using the higher frequency. Using pedigrees as independent units instead of nuclear families yielded nonsignificant results; given the small number of pedigrees, this was expected.

**Table 3:** *P*-values corresponding to the true causal genes using Q1 as phenotype

Chromosome	Gene	1% cutoff		3% cutoff	
		
		Nuclear families	Pedigrees	Nuclear families	Pedigrees
1	*ARNT*	0.441	0.301	0.450	0.406
1	*ELAVL4*	0.447	0.347	0.952	0.948
4	*KDR*^a^	0.03	0.09	0.229	0.092
4	*VEGFC*^a^	0.009	0.317	0.009	0.317
5	*FLT4*	0.314	0.299	0.319	0.304
6	*VEGFA*^a^	0.0002	0.122	0.002	0.156
13	*FLT1*^a^	0.076	0.128	0.0003	0.024
14	*HIF1A*	NA	NA	0.317	0.317
19	*HIF3A*	0.508	0.466	0.638	0.609

^a^ Gene that has polymorphic causal SNPs. The other five causal genes (not marked by superscript a) cannot be identified in our method because there were no causal SNPs corresponding to those genes in the sample.

**Figure 1:**
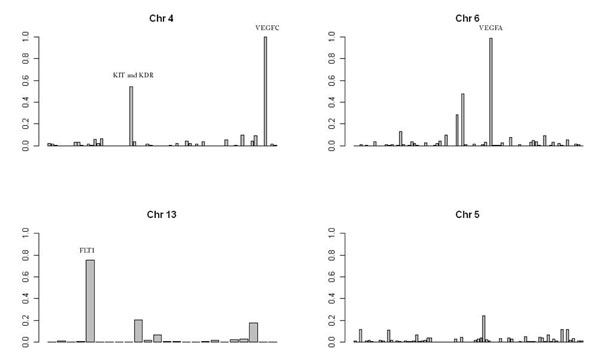
**Detection rates from modified FBAT for all genes on chromosomes 4, 5, 6, and 13.** Each bar in the graphs represents the percentage of times that the gene was significant (*p* < 0.05) in the 200 replicates. True-positive disease genes are labeled. Of note, the *KIT* locus on chromosome 4, frequently detected as a false positive, is in close proximity (394 kb) to the disease-causing *KDR* locus.

To estimate the FBAT statistic’s true- and false-positive rates, we ran our method on the 200 individual phenotype replicates and reported the proportion of times a gene was declared significant (at *p* < 0.01). As can been seen in Table 4, the FBAT has high power to detect association for three of the four polymorphic causal genes: power approaches 1 for *VEGFA* and *VEGFC*, regardless of allele frequency cutoff, whereas power varies by allele frequency cutoff for *FLT1*. Power is poor for *KDR*, regardless of cutoff. Among genes that were modeled as disease causing but for which random sampling resulted in the absence of polymorphic rare variants in our data sets, the false-positive rates are low. Two related genes, *HIF1A* and *HIF3A*, have false-positive rates of 0, and the other three genes have rates no higher than 0.02, suggesting high test specificity (not shown). However, a more comprehensive assessment of all genes reveals a substantially higher false-positive rate. Figure 1 graphs the detection rates for all genes on chromosomes 4, 5, 6, and 13. We found several genes that seem to have high rates of detection despite not being associated with the trait. Most notable are *PCDHGA2* (rate = 0.245), *PSMB8* (rate = 0.475), and *TRPC4* (rate = 0.205). The high false-positive rate for *KIT* can be explained by its close proximity to *KDR*.

**Table 4:** True-positive rates corresponding to the true causal genes using Q1 as phenotype (estimated from the 200 replications provided in the GAW17 data set)

Chromosome	Gene	1% cutoff	3% cutoff
4	*KDR*	0.085	0.035
4	*VEGFC*	0.995	1
6	*VEGFA*	0.995	0.990
13	*FLT1*	0.075	0.775

## Discussion

Rare variants are likely to be private to one or a limited number of families. As a consequence, it is likely that the genetic liability conferred by rare variants will exhibit pronounced genetic heterogeneity, with different individual contributions from numerous variants. It is well recognized that model misspecification, including failure to consider allelic heterogeneity, can severely limit disease-gene mapping efforts. It therefore follows that gene-mapping efforts that focus on rare variants accommodate this reality. In our study, aggregating linkage statistics across all pedigrees yielded negative results, whereas modeling linkage within individual pedigrees performed well. So linkage analysis shows some promise in analyzing rare variants given sufficiently large pedigrees.

The modified FBAT is promising. It correctly identifies causal genes that contain polymorphic SNPs in the family sample. However, we found that there were considerable false positives; many factors could be responsible for the high false-positive rates, for example, failure to adjust for multiple testing, linkage disequilibrium between causal and noncausal SNPs, incorrect variance estimation, lack of normality resulting from the restriction to rare variants, and the method used to simulate the replications.

With regard to variance estimation, there are only 8 pedigrees and 194 nuclear families, so differences in the two approaches to computing the variance are to be expected. In study designs often seen in actual samples, these differences may not be so important, but clearly, better approaches are needed. Some limited examination of the sensitivity of the false-positive rate suggests that the use of only rare variants does not have a major impact. Furthermore, the simulation structure of the family-based sample makes it difficult to evaluate performance of any family-based methods. First, many of the true causal SNPs are not polymorphic in the family-based sample, making it impossible for both linkage and association analyses to identify the causal genes with those variants. Second, for the proposed family-based methods the random variable is the transmission of genotype. Hence the simulated replicates of phenotypes cannot be used to appropriately evaluate power or validity of such methods.

Further research should investigate possible approaches to extend the proposed association test using variable thresholds for identifying rare variants and using available pathway information. Another issue that can be addressed in future research is the assumption that all rare variants act in the same direction, affecting the disease risk; potential ways to address the violation of such an assumption in the context of our method should be tested.

## Conclusions

Linkage, stratified by pedigree, provides a promising method for identifying rare variants, provided that pedigrees are large. The modified FBAT approach also suggests that it is a promising approach, but the false-positive rates need to be addressed. Although not attempted here, a promising scenario may be to combine the two approaches, using linkage to screen genes or regions and then using the FBAT for testing selected regions. Given the scale of large-scale sequencing, this approach not only may be more powerful but may also provide substantial cost savings. Finally, methods for evaluating power and type I error for linkage and transmission testing need to be designed differently to provide valid estimates for those tests.

## Competing interests

The authors declare that there are no competing interests.

## Authors’ contributions

W-K Yip performed the initial cleaning of the data set and linkage analysis; GD developed and applied the novel FBAT statistics for the fine mapping analysis. BARy and NL supervised the project.
